# Comparison of silver and gold nanoparticles green synthesis by *Artemisia annua* hairy root extracts

**DOI:** 10.1242/bio.061739

**Published:** 2025-03-19

**Authors:** Taisa Bohdanovych, Pavlo Kuzema, Viktor Anishchenko, Volodymyr Duplij, Maksym Kharchuk, Viktoriia Lyzhniuk, Anatolij Shakhovsky, Nadiia Matvieieva

**Affiliations:** ^1^ Institute of Cell Biology and Genetic Engineering of NAS of Ukraine, Kyiv, Ukraine, 03143; ^2^ Chuiko Institute of Surface Chemistry of NAS of Ukraine, Kyiv, Ukraine, 03164; ^3^ L.M. Litvinenko Institute of Physical-Organic Chemistry and Coal Chemistry of NAS of Ukraine, Kyiv, Ukraine, 02155; ^4^ D.K. Zabolotny Institute of Microbiology and Virology of NAS of Ukraine, Kyiv, Ukraine, 03680; ^5^ Kyiv National University of Technologies and Design, Kyiv, Ukraine, 01011

**Keywords:** *Artemisia annua*, Hairy roots, Silver nanoparticles, Gold nanoparticles, Green synthesis

## Abstract

The green synthesis of metal nanoparticles (NPs) has garnered significant attention due to its simplicity, cost-effectiveness, and environmental sustainability. Gold NPs (AuNPs) and silver NPs (AgNPs) are widely employed across various industries, agriculture, and medicine owing to their unique physicochemical properties. This study explores the feasibility of synthesizing metal NPs through green methods using ethanolic (70%) extracts from *Artemisia annua* hairy roots. These extracts were found to contain reducing agents, primarily phenolic compounds, as identified by HPLC and MALDI-MS analyses. The phenolic compounds included hydroxybenzoic acids (e.g. p-coumaric and gallic acids) and hydroxycinnamic acids (e.g. caffeic acid and its derivatives such as chlorogenic, dicaffeoylquinic, and rosmarinic acids). The synthesis and structural characteristics of AuNPs and AgNPs were systematically compared. AgNPs formed a stable colloidal solution over extended periods, while AuNPs exhibited instability due to significant NP aggregation and precipitation. Furthermore, the photocatalytic activities of these NPs in the degradation of Methylene Blue were evaluated. AuNPs demonstrated substantial photocatalytic activity, whereas AgNPs exhibited negligible catalytic effects. This study highlights the potential and limitations of *A. annua* hairy root extracts in the biosynthesis of AuNPs and AgNPs, providing insights into their structural and functional differences.

## Teaser

**Summary:** The composition of hairy root extracts greatly affects green synthesized gold and silver nanoparticles' features, the process of their synthesis and their stability.

## INTRODUCTION

*Artemisia annua* (sweet wormwood) has become one of the flagships of all plants due to its fame after Chinese scientist Tu Youyou received the Nobel Prize in 2015 for the discovery of artemisinin. Many scientists focus currently on artemisinin (Sutherland et al., 2021; Wani et al., 2021; Feng et al., 2022; Zhu and Zhou, 2022; Stead et al., 2023), as it is a drug used to fight malaria. Studies on susceptibility and sensitization of *Plasmodium falciparum* (malaria parasite) to the artemisinin family of antimalarial drugs are being performed, possible ways to enhance artemisinin content in plant material, as well as the research on the potential antidiabetic effect of artemisinin. Thus, there are many articles that study the practical application of sweet wormwood (Fuzimoto, 2021; Al-Khayri et al., 2022; Guo et al., 2024). Its antiviral activity against SARS-CoV-2, possible use for cancer treatment, anti-inflammatory and antioxidant activities were researched.

Thus, *A. annua* plants are characterized by a complex of biological activities. This is possible due to the presence in these plants of a large number of compounds that are known for their bioactivity. This wormwood synthesizes essential oils, mono- and sesquiterpenes, flavonoids and other polyphenolic compounds as well (Ko et al., 2020; Hong et al., 2023; Shinyuy et al., 2023). Therefore, sweet wormwood can serve as a source of a whole complex of chemical compounds with antioxidant activity and other properties.

Based on the fact that this plant has polyphenols with reactive groups with reducing activities, there is a possibility of using *A. annua* extracts not only for different diseases treatment but also to reduce metals for nanoparticle (NP) synthesis. For instance, Aghajanyan et al. (2020) used aqueous extract of leaf powder and obtained silver NPs. Wang et al. (2020) used sweet wormwood stem aqueous extract to synthesize zinc oxide NPs. Karimi et al. (2024) initiated formation of copper oxide NPs with aqueous extract of *A. annua*, while Anand Mariadoss et al. (2022) used such extracts for selenium obtaining NPs. NPs obtained via green synthesis are of interest in pharmacology and biomedical engineering, as they have antiviral and antioxidant properties with low cytotoxicity and acceptable biocompatibility (Dehghani et al., 2023), as well as potent antifungal (Sharma et al., 2022; Ali and Abdallah, 2022), antimicrobial (Elemike et al., 2018) and antibiofilm (Moulavi et al., 2019), anticancer (Mikhailova, 2022; Mousavi et al., 2018), and apoptosis-inducing effects (Bordoni et al., 2021; Mousavi et al., 2018).

The green synthesis method of obtaining NPs has significant advantages over chemical and physical synthesis (Radulescu et al., 2023). It avoids the use of toxic agents that can produce potential risk to environment. Moreover, green synthesis can proceed in moderate pH values and with the use of fewer purification steps. Other benefits include the lower cost of NPs production, ambient temperature and pressure for the synthesis process (Singh et al., 2023). Additionally, this method can find the application for obtaining bimetallic and trimetallic NPs (Idris and Roy, 2024; Idris et al., 2025, 2024; Gupta et al., 2024).

The study of the process of green synthesis of metal NPs has recently received considerable attention, which is connected with the simplicity and availability of their synthesis and the possibility of their practical application, in particular, in medicine. Extracts from various plant species are used now for these purposes. At the same time, since the composition of the extract significantly affects the process of green synthesis of metal NPs, special attention should be paid to the selection of plants for obtaining extracts, taking into account the tasks regarding certain characteristics of NPs. Unfortunately, little attention has been paid to these issues so far. This is evidenced by the lack of publications regarding the comparative characteristics of the synthesis of NPs of various metals depending on the chemical composition of the extracts used.

Typically, extracts from wormwood leaves were used for the green synthesis of NPs (Basavegowda et al., 2014; Sundararajan and Ranjitha Kumari, 2017; Elemike et al., 2018; Mousavi et al., 2018; Aghajanyan et al., 2020; Ali and Abdallah, 2022; Bordoni et al., 2021; Ghramh et al., 2021), as they contain a complex of chemically active compounds. In addition, an extract from wormwood callus cells was also used for this purpose (Xia et al., 2017). However, the hairy roots of *A. annua* have not yet been used to obtain metal NPs outside our institution. This research is novel and has a number of advantages over using extracts from plant leaves. Such advantages arise from the specific characteristics of hairy roots and their possibly significantly higher content of chemically active compounds.

Hairy roots are transgenic roots that derive from the wounded plants co-cultivated with Rhizobium (Agrobacterium) rhizogenes. These soil bacteria can infect plants and transfer the part of their genome (T-DNA with *rol* genes) to the plants. Hairy roots have a number of advantages, including the ability of cultivation for unlimited time, synthesis of plant-specific compounds in an amount that significantly exceeds that of the mother plants, and synthesis of compounds of mammalian or bacterial origin using transferred genes (Tavassoli and Safipour Afshar, 2018; Mohaddab et al., 2022; Cardon et al., 2019). This method of genetic engineering allows the metabolism of cells to be changed and stimulates the synthesis of specific plant components or compounds that are not typical for plants. Bacterial *rol* genes serve as those ‘boosters’ of secondary metabolism, allowing to obtain more compounds with reducing activity, such as polyphenols, simple phenol and hydroxybenzoic acids and their derivatives (Spiegel et al., 2020; Kalinowska et al., 2021; Naróg and Sobkowiak, 2023). Thus, it may be more beneficial to use the extracts of hairy roots to reduce metals, than to use the extracts of non-transformed plants.

Earlier we have succeeded to obtain silver NPs (Kobylinska et al., 2020), magnetite (Fe_3_O_4_) and cobalt ferrite (CoFe_2_O_4_) NPs (Kobylinska et al., 2021) from sweet wormwood hairy roots. The aim of this study was to evaluate and compare the characteristics of the NPs of different metals (AuNPs and AgNPs) obtained using *A. annua* hairy root extracts. This study is the first to characterize the synthesis of NPs of different metals in particular such as AuNPs and AgNPs using the same extracts from hairy roots.

## RESULTS

### Reducing activity of *A. annua* extracts

**Fig. 1.**
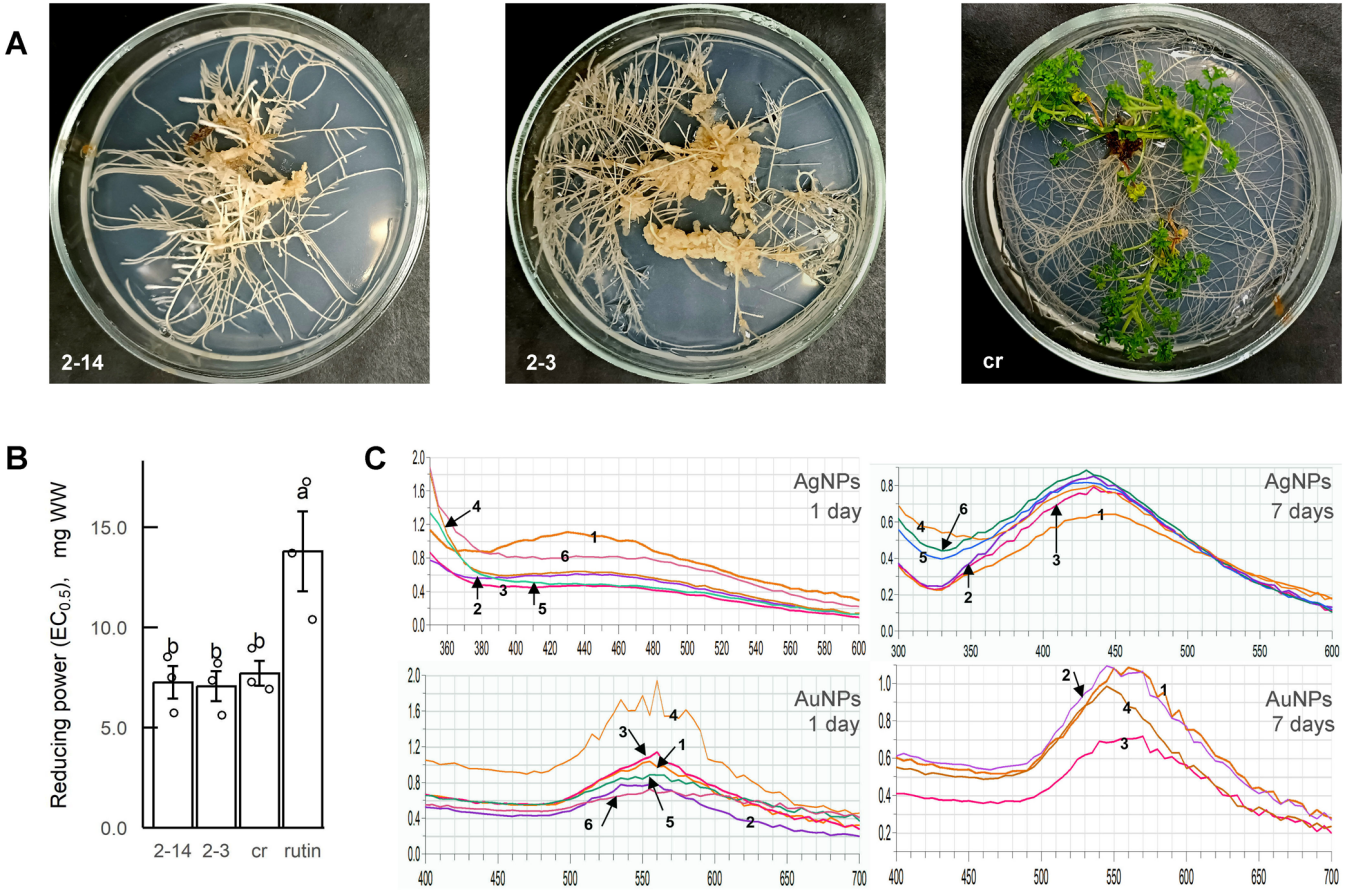
**Silver and gold NPs (AgNPs and AuNPs) obtaining using *A. annua* extracts.** (A) *A. annua* control plants (cr) and hairy root lines (2-3 and 2-14) cultivated *in vitro*. (B) Reducing activity of the standardized extracts (1 mg RE/ml) of *A. annua*; *n*=3 experimental repeats; mean±s.e.; tested on normality by the Shapiro–Wilk test and homogeneity of variance by Levene's test; comparisons were performed by one-way ANOVA, followed by the Tukey HSD test; *P*<0.05. (C) UV-Vis spectra of AgNPs and AuNPs obtained from *A. annua* extracts in one (not diluted) and 7 days. 7 days: AgNPs were diluted six times; AuNPs – samples 1, 2, 3 were diluted three times after green synthesis by *A. annua* extracts from control roots (sample 1-40 µl/1 ml of metal solution; sample 4-80 µl/1 ml), hairy root line 2-3 (sample 2-40 µl/1 ml; sample 5-80 µl/1 ml), and hairy root line 2-14 (sample 3-40 µl/1 ml; sample 6-80 µl/1 ml); * – AuNPs samples 5 and 6 were not studied in 7 days because of their partial sedimentation.

Since the original extracts from the roots of control *A. annua* plants and two lines of hairy roots (2-3 and 2-14) had different concentrations of flavonoids, the dry extracts were dissolved in 70% ethanol to a concentration of 1 mg/ml in rutin equivalent to standardize them. Analysis of the reducing activity of the extracts obtained in this way revealed no differences between them (Fig. 1B). The activity of the extracts was higher than the same activity of rutin solution (1 mg/ml).

### HPLC and MALDI MS analyses of extracts

**Fig. 2.**
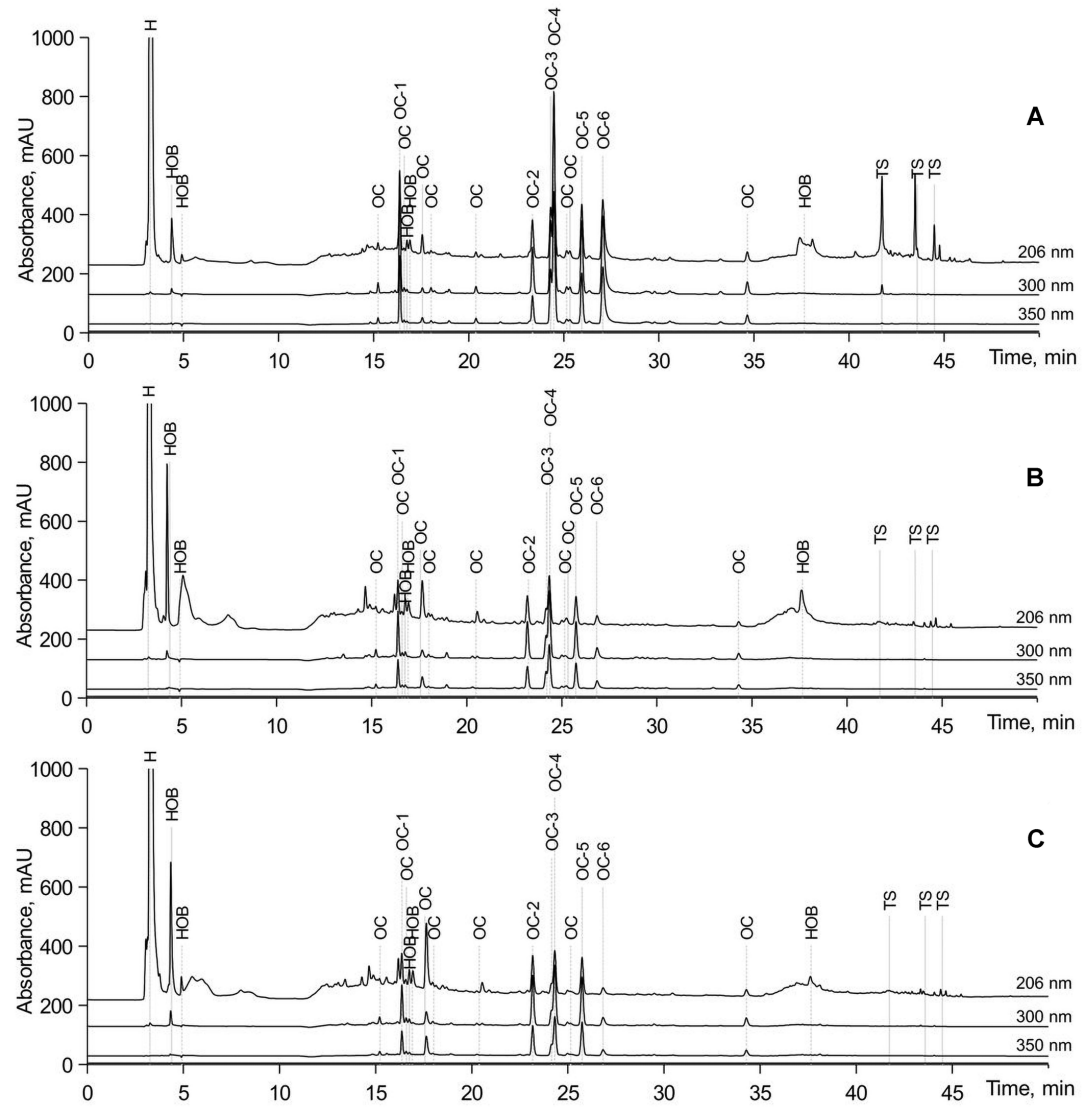
**Representative chromatograms of *A. annua* extracts.** (A) Control roots, (B) transgenic line No. 2-3, and (C) transgenic line No. 2-14. H, strongly hydrophilic compounds; HOB, simple phenols and derivatives of hydroxybenzoic acids; TS, terpenoids; OC, derivatives of caffeic acid.

It was previously established that *A. annua* plants synthesize a number of bioactive compounds including sesquiterpenoids, flavonoids, coumarins, lipids, phenolics, purines, steroids, triterpenoids, aliphatics, and artemisinin (Anibogwu et al., 2021; Ferreira et al., 2010; Sanjay Kumar Rai et al., 2021). In our study according to the results of chromatographic analysis, the extracts used in the experiment contained the following classes of organic compounds: strongly hydrophilic compounds (H); simple phenols and derivatives of hydroxybenzoic acids (HOB); terpenoids (TS); derivatives of caffeic acid (OC). Among the derivatives of caffeic acid, chlorogenic acid (OC-1) was identified (Fig. 2). Other derivatives of caffeic acid with a content of more than 5% of the total amount of OC were also marked with separate numbers (OC-2-OC-6). The content of simple phenols and derivatives of hydroxybenzoic acids in the control roots was lower than in transgenic one. At the same time content of other organic compounds including chlorogenic acid and derivatives of caffeic acid (OC-1, OC-3-OC-6) detected in the control roots exceed this parameter in the hairy root lines. Table 1 shows the studied content of various compounds in the extracts. The content of OC and OC-1 to OC-6 is given in terms of chlorogenic acid, and the content of H, HOB and TS in terms of gallic acid.

**Table 1.** Content of organic compounds (μg/ml) in extracts from *A. annua* control roots (sample 1) and hairy root lines 2-3 and 2-14 (samples 2 and 3, respectively)

Sample	Content of organic compounds (μg/ml)									
	H	HOB	TS	OC	OC-1	OC-2	OC-3	OC-4	OC-5	OC-6
С	560	88	10	68	93	57	79	266	94	154
2-3	598	150	8	68	74	46	53	186	72	127
2-14	694	125	6	51	35	66	15	88	67	16

*Peak designations: H, hydrophilic compounds; HOB, simple phenol/hydroxybenzoic acids and their derivatives; ОC, ОС-1-ОС-6, derivatives of caffeic acid; OC-1, chlorogenic acid; TS, terpenoids.

The results of negative- and positive-ion MALDI mass spectra analysis for the samples of *A. annua* root extracts are shown in Tables 2 and 3. It can be seen that the majority of signals in the mass spectra belong to the ions attributed to phenolic (hydroxybenzoic and hydroxycinnamic) acids and their derivatives, in particular to caffeic acid and its derivatives (chlorogenic, dicaffeoylquinic, rosmarinic acid). This is consistent with the HPLC data on predominance of peaks related to phenolic acids and their derivatives. The most intense signals in the mass spectra were assigned to ions belonging to such phenolic acids as p-coumaric and gallic acid, as well as to other polyphenols (coumarins) and other classes of compounds, such as quinic acids, carboxylic acids (including amino acids), benzenoids and terpenoids. The signals related to carbohydrates, simple phenols, flavonoids, and stilbenes were also present.

**Table 2.** Results of negative-ion MALDI MS studies on the composition of *A. annua* root extracts using the THAP matrix (1) the extract of the control roots; (2 and 3) extracts of transgenic roots 2-3 and 2-14

m/z	Attributed ion(s)	Assigned compound(s)	Relative peak intensity (%) in the mass spectrum*		
			1	2	3
103.0	[C3H4O4-H]–	Tartronate semialdehyde	20	7	6
133.0	[C4H6O5-H]–	Malic acid	86	37	55
163.0	[C9H8O3-H]–	p-Coumaric acid^‡^/caffeic aldehyde	100	100	100
179.1	[C9H8O4-H]–	Caffeic acid^‡^	34	14	14
191.1	[C7H12O6-H]–	Quinic acid^‡^	67	6	8
196.0	[C5H11NO3S2-H]–	S,S-(2-Hydroxyethyl)Thiocysteine	---	6	5
198.0	[C4H10NO6P-H]–	Phosphonothreonine	9	---	---
229.0	[C5H11O8P-H]–	Pentose phosphate(s)	15	12	11
250.1	[C12H13NO5-H]–	N-Feruloylglycine	7	5	---
252.1	C12H12O6–	Caffeoylglycolic acid methyl ester	---	---	7
268.0	[C7H12NO8P-H]–	N-Acetyl-L-glutamyl 5-phosphate	14	36	38
290.1	[C11H17NO6S-H]–	Hawkinsin	33	19	16
353.1	[C16H18O9-H]–	Chlorogenic acid^‡^	23	---	3
391.1	[C18H16O10-H]–	Cimicifugic acid H	15	---	---
515.2	[C25H24O12-H]–	Dicaffeoylquinic acid^‡^	8	---	---

*For the analyte peaks with not less than 3% intensity relative to the one of highest intensity.

^‡^Found in *Artemisia* species according to the data from literature (Bordean et al., 2023; Ekiert et al., 2022).

**Table 3.** Results of positive-ion MALDI MS studies on the composition of *A. annua* root extracts using the THAP matrix (1) the extract of the control roots; (2 and 3) extracts of transgenic roots 2-3 and 2-14

m/z	Attributed ion(s)	Assigned compound(s)	Relative peak intensity (%) in the mass spectrum*		
			1	2	3
115.0	[C2H4O3+K]+	Glycolic acid	38	18	35
116.1	[C5H9NO2+H]+	Proline	22	19	34
140.0	C11H8•+	2,4-Pentadiynylbenzene^‡^	100	100	100
147.1	[C9H6O2+H]+	Coumarin^‡^	29	6	16
154.0	C7H6O4•+/C12H10•+	Dihydroxybenzoic acid/acenaphthene^‡^	11	---	7
156.1	C12H12•+ /	1,4-Dimethylnaphthalene^‡^ /	10	---	7
	[C8H13NO2+H]+	Scopoline^‡^			
168.0	C8H8O4•+	Vanillic acid^‡^	21	8	---
169.0	[C9H6O2+Na]+	Coumarin^‡^	13	23	39
171.0	[C7H6O5+H]+	Gallic acid^‡^	14	---	28
175.1	[C10H16+K]+	Pinene/limonene/camphene/carene/ocimene/terpinene/terpinolene/sabinene/artemisia trien^‡^	48	15	17
185.0	[C9H6O2+K]+	Coumarin^‡^	91	13	35
206.0	[C4H9NO2S2+K]+	(2R)-2-amino-3-(methyldisulfanyl)propanoic acid	11	16	16
209.0	[C7H6O5+K]+	Gallic acid^‡^	11	10	56
219.0	[C9H8O4+K]+	Caffeic acid^‡^	21	---	---
223.0	[C11H10O5+H]+	Isofraxidin/tomentin^‡^	31	16	29
245.0	[C11H10O5+Na]+	Isofraxidin/tomentin ^‡^	4	16	26
330.0	[C11H17NO6S+K]+	Hawkinsin	40	8	17
368.0	C17H20O9•+	Feruloylquinic acid^‡^/caffeoylquinic acid methyl ester	10	---	13
369.1	[C17H20O9+H]+	Feruloylquinic acid^‡^/caffeoylquinic acid methyl ester	7	---	---
370.0	C15H14O11•+	2-O-Caffeoylhydroxycitric acid	---	---	11
371.1	[C15H14O11+H]+	2-O-Caffeoylhydroxycitric acid	7	---	---
381.0	[C15H18O9+K]+	Caffeoylglucose/caffeoyl-β-D-glucopyranoside	21	14	13
539.1	[C25H24O12+Na]+	Dicaffeoylquinic acid^‡^	---	---	15
543.1	[C21H28O14+K]+	Caffeic acid glycoside(s)	11	---	8
777.3	[C34H42O18+K]+	Glycoside(s) of flavone(s)/flavonols(s)	18	32	35
801.3	C38H41O19•+	Malvidin glycoside(s)	9	15	22

*For the analyte peaks with not less than 3% intensity relative to the one of highest intensity.

^‡^Found in *Artemisia* species according to the data from literature (Anibogwu et al., 2021; Bordean et al., 2023; Ekiert et al., 2022).

The process of NPs initiation depended on the content of plant extracts in the reaction mixture (Fig. S1). In particular, the color of the mixture did not change in case when small amount of the extracts (5-10 μl/1 ml) was added to HAuCl4 solution. Thus, in the next study we used the higher concentrations both for silver and gold NPs obtaining.

### UV-Vis spectra of NPs colloid solutions

AgNPs and AuNPs NPs were characterized by specific optical absorption spectra in UV-visible region called surface plasmon resonance. The formation of AgNPs and AuNPs was monitored by the specific absorption band in the range of 400-450 nm and 500-550 nm, respectively (Fig. 1C). As it can be seen, the process of formation of AgNPs was not instantaneous and continued for at least a week, as there were significant changes in the UV-Vis spectra 7 days after the initiation. The same effect was studied in the process of both silver and gold NPs synthesis.

**Fig. 3.**
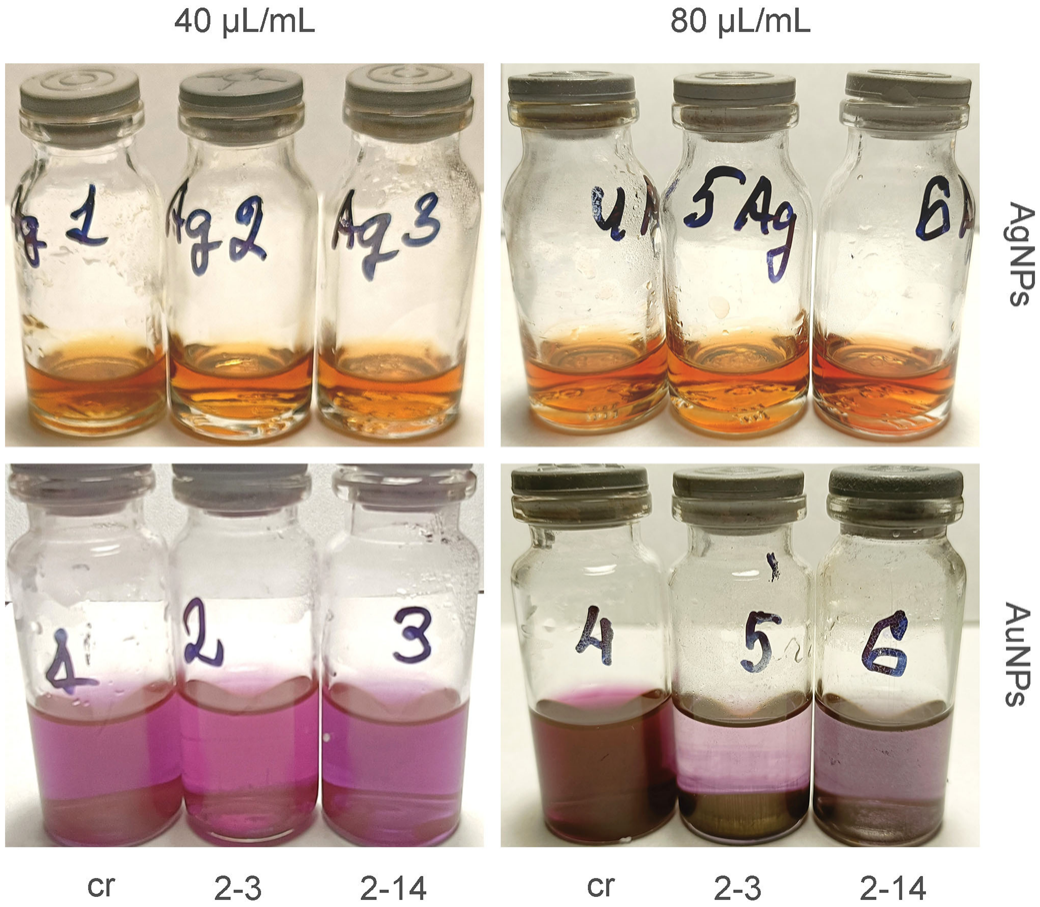
**Images of AgNPs and AuNPs colloid solutions obtained from *A. annua* extracts.** Silver and gold NPs solutions 7 days after initiation: extracts of the control roots (cr), hairy root lines 2-3 and 2-14 used in the ratio of 40 µl/1 ml and 80 µl/1 ml for green synthesis of NPs.

It should be noted that the solutions of AgNPs remained stable without sedimentation during their storage (Fig. 3). At the same time, the obtained solutions of AuNPs had a tendency to sedimentation a week after their initiation (Fig. 3). Therefore, it was not possible to obtain the correct spectra of these samples within 7 days of NPs formation (Fig. 1C, AuNPs 7 days). The higher was the ratio of extract/HAuCl_4_ solution, the faster was the process of NPs sedimentation.

### Fourier-transform infrared (FTIR) analysis

**Fig. 4.**
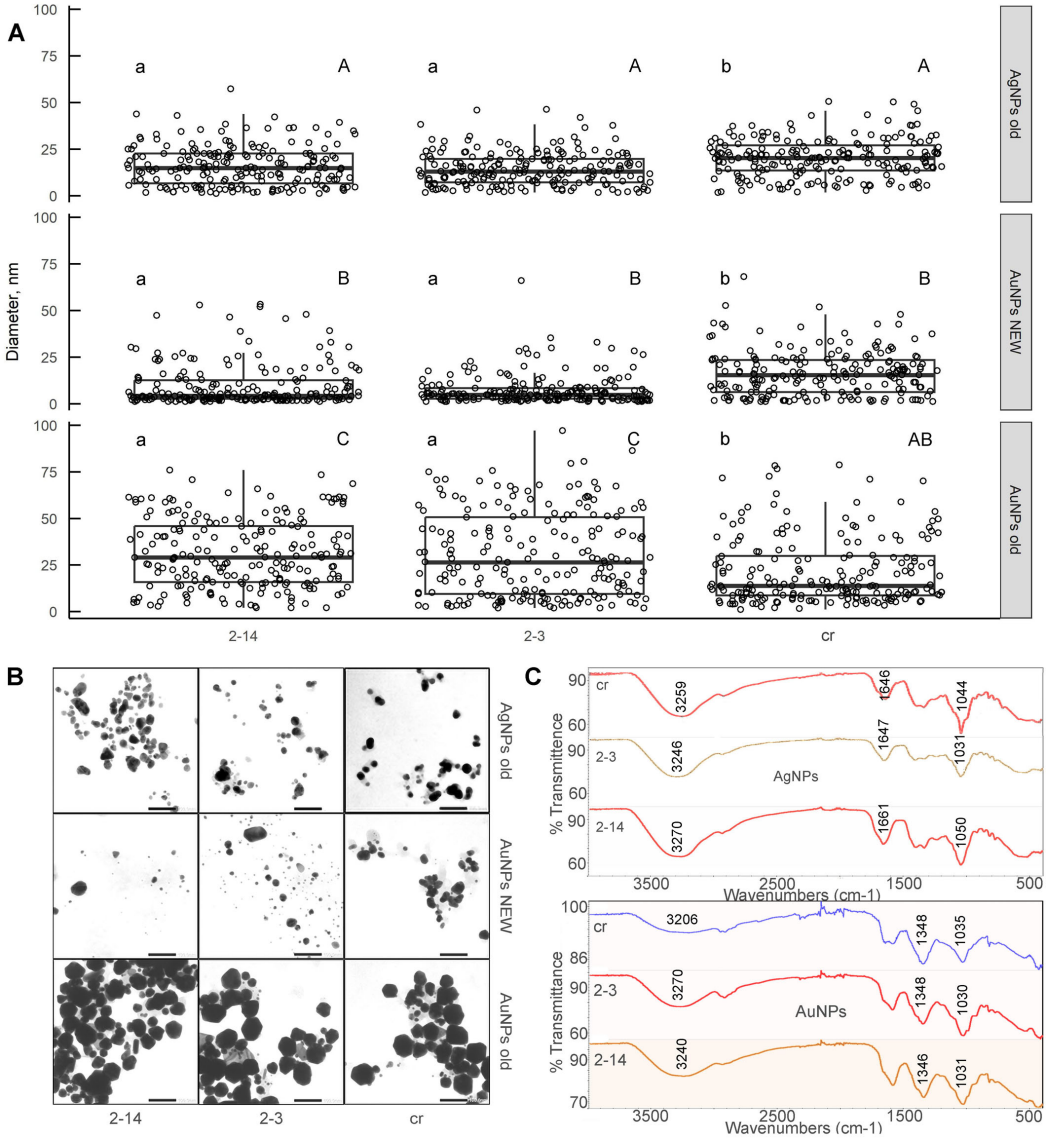
**Characteristics of NPs.** (A) Size distribution of AgNPs and AuNPs obtained by green synthesis using *A. annua* control roots (cr), hairy root lines 2-3 and 2-14, respectively. NEW – on the day of NP initiation, old – in 14 days after NPs formation. For all variables with the same letter, the difference between the medians is not statistically significant. Lowercase letters indicate comparison of sizes of NPs obtained by three different extracts; uppercase letters indicate differences between NPs (gold and silver) obtained by the same extract; *n*=200 experimental repeats; mean±s.e.; tested with Kruskal–Wallis rank sum test, followed by the corresponding multiple comparison test from the pgirmess package; *P*<0.05. (B) TEM images of AgNPs and AuNPs synthesized by *A. annua*. Scale bars: 100 nm. (C) FTIR spectra of silver and gold NPs.

The AuNPs synthesized by *A. annua* control root and hairy roots extracts yielded bands at 3206, 2953-2918, 2323-1989, 1590, 1348, 1034-1029, 823, and 548-525 cm^−1^ in FTIR study. Samples synthesized using hairy root extracts No. 2-3 and 2-14 had bands at 3239-3270 cm^−1^ as well (Fig. 4C, more detailed information is in Fig. S6,7). The AgNPs yielded bands at 3246-3292, 2940-2928, 1646-1659, 1238-1416, 1050-1231, 922-776 cm^−1^ (Fig. 4C, more detailed information is in Fig. S2-4). The FTIR spectroscopy of NPs indicated the involvement of some functional groups in the reduction of gold and silver ions and NPs formation. In particular, the broad peak at nearly 3229-3290 cm^−1^ in the NPs spectra can be assigned to -OH group or stretching of N-H. The peak at 2929-2940 cm^−1^ indicates –C–H stretching. The absorption band at about 1643-1661 cm^−1^ can be assigned to the stretching vibration of –C=O. The observed peaks at nearly 1580 cm^−1^ can be assigned to the stretching vibration of the carboxylate –COO− groups. The band at 1386-1404 cm^−1^ corresponds to –CH_3_ groups (Elavazhagan and Arunachalam, 2011; Rajeshkumar, 2016; Han et al., 2000; Alegria et al., 2018; Ahmad et al., 2019).

### Transmission electron microscopy (TEM) analysis

AgNPs samples were stable even after long-term incubation. This is evidenced by the preservation of the transparency and color of the colloidal solution, as well as by the results of spectroscopic analysis in the 300-700 nm range. According to TEM analysis (Fig. 4B) NPs were mostly less than 20 nm in diameter (used by the extract from the control roots – 48%, by hairy root No. 2-3–76%, by hairy root No. 2-14–65% in 14 days) (Fig. 4A). There was no sediment in the colloid solutions even when it was stored for more than 6 months. No drastic conglomeration or conglutination was visualized. The shapes of NPs of all samples were more spherical and barrel-like.

There was a difference in size distribution of AuNPs, that were freshly obtained (1 day from the initiation) and that were stored for 2 weeks (Fig. 4B). Freshly obtained solutions of AuNPs were truly colloid, as there was no sediment. The percentage of a small (up to 20 nm) NPs was 67%, 93%, and 85% when the extracts from the control roots, root line 2-3 and line 2-14 were used, respectively (Fig. 4A). Conglomeration and conglutination of particles were mostly absent. Maximal sizes of these NPs were 50-60 nm. Contrary to that, solutions stored for 2 weeks showed the presence of high number of conglomerated particles that were more than 50 nm in diameter with the maximal sizes 80-90 nm. Interestingly enough, such conglutination was more visible in samples obtained via green synthesis using hairy root extracts. In these samples the percentage of small NPs (less than 20 nm) was 43% and 34% (lines 2-3 and 2-14, respectively). Such a significant increase in the size of NPs can be the cause of sedimentation under the condition of insufficient concentration of stabilizing agents in the used extracts. Apart from the size distribution, the samples significantly differed in the shape as well. NPs of samples stored for 2 weeks had the variety of shapes: triangular, pyramidal, spherical, and icosahedron-like, with the last type being the most abundant. In freshly obtained samples all these types of shapes were visualized as well; however, the facets of icosahedrons were more smoothed. Specificity of the shape may be one of the reasons of AuNPs instability. In particular, in the model conditions spheres were found to be much more stable than other shapes of AuNPs (Carone et al., 2023). Authors found that the shape plays the major role in the stability of NPs colloidal solution: the presence of sharp tips may be the source of instability.

### Photocatalysis of Methylene Blue (MB) solution by NPs

**Fig. 5.**
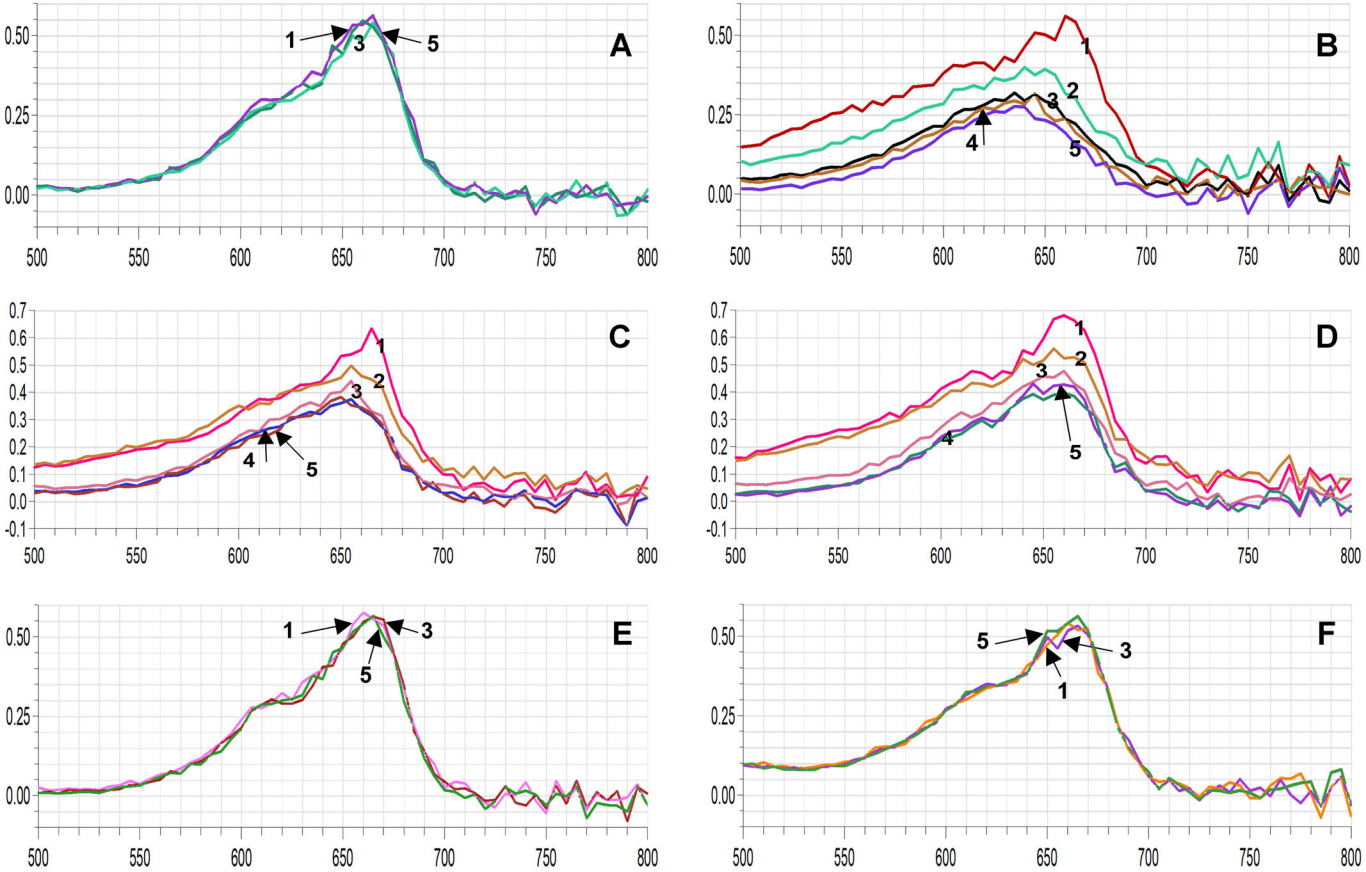
**Photobleaching of MB by AgNPs and AuNPs obtained after green synthesis using *A.*** ***annua*** **extracts**. (A) UV-Vis spectra of blank MB, (B) MB+AuNPs from control roots, (C) MB+AuNPs from hairy root line 2-3, (D) MB+AuNPs from hairy root line 2-14; (E) MB+AgNPs from control roots; (F) MB+AgNPs from hairy root line 2-3; 1-10 min after the start of UV irradiation, 2-15 min, 3-30 min, 4-45 min, 5-60 min.

The photodegradation of MB stimulated by colloid solutions of different NPs, such as WO_3_NPs (Yin et al., 2021), AuNPs (Maliszewska et al., 2021; Suvith and Philip, 2014), AgNPs (Suvith and Philip, 2014) is a well-known process. In our study the effects of green synthesized AuNPs and AgNPs were compared. The characteristic absorption peak of MB around 660 nm (León et al., 2016; Maliszewska et al., 2021) was observed at the start of each reaction and throughout the 1-hour irradiation of blank MB solution (Fig. 5). This peak significantly decreased in all experimental samples when AuNPs were added to the reaction mixture (Fig. 5B-D) and almost didn't change in blank MB solution during the experimental time (Fig. 5A). Contrary to these results, AgNPs in this reaction exhibited negligible catalytic effects, thus almost no change in the characteristic absorption peak was observed (Fig. 5E,F).

In all samples containing AuNPs the fastest photobleaching occurred in the first 15 min. Then, with each time interval the bleaching proceeded less and less intensively, with almost no change in absorption after 45 min of irradiation. Therefore, the role of gold NPs, obtained from *A. annua*, as an electron transfer catalyst is the most prominent only shortly after the start of UV irradiation.

## DISCUSSION

Previously, extracts from *A. annua* were used to obtain metal NPs. For example, water extract from the leaves of hydroponically grown plants (leaf powder) was used to obtain silver NPs. These NPs were 20-90 nm sized range and had spherical shape (Aghajanyan et al., 2020). Xia et al. (2017) extracted *A. annua* fresh calli in sterile distilled water and used this extract for AgNPs greensynthesis. In this study NPs were mostly spherical with the size of 2.1 to 45.2 nm and average size of 10.9 nm. However, larger NPs (up to 100 nm) were also obtained using *A. annua* extracts (Ghramh et al., 2021). AuNPs were prepared earlier using leaf extract of *A. annua* (Basavegowda et al., 2014; Boomi et al., 2020). AuNPs had triangular and spherical shapes with sizes ranging from 15 to 40 nm. We would like to note that in all these studies, only plants or callus cultures were used for extracting. In addition, the above publications lack information on the chemical composition of the used extracts, as well as data on the stability of the obtained NPs. At the same time, thanks to genetic transformation and the incorporation of bacterial *rol* genes to plant genome, such transgenic roots can have the specificity of secondary metabolism. Thus, the content of bioactive compounds, including those with reducing properties (for example, phenolic compounds) and those that can be NP's stabilizers, can vary significantly.

Phenolic compounds are native for different plant species (Zhang et al., 2022). Owing to their specific structure, they are chemically active and protect plants against reactive oxygen species and allow plant to minimize negative effect of stress factors (Kumar et al., 2023). These plant-derived chemicals with functional groups are widely used in medicine as potent antioxidants (Dai and Mumper, 2010; Sathishkumar et al., 2018). We paid attention to the *A. annua* root extracts with different phenolics because these components play role as both reducing agents and stabilizers in the process of NPs synthesis (Amini and Akbari, 2019). That is why we used the *A. annua* root extracts for AgNPs and AuNPs green synthesis.

Thus, according to the above research results, the extracts from *A. annua* hairy roots and roots of the control plants contained a complex of compounds that are chemically active and have reducing activity. For example, such compounds as polyphenols, terpenoids, flavonoids, gallic, caffeic, and chlorogenic acids were applied earlier for silver and gold NPs initiation (Wang et al., 2007; Noh et al., 2013; Mittal et al., 2014; Guo et al., 2015; Hwang et al., 2015; Seo et al., 2017; Sundararajan et al., 2017).

Since extracts from all root samples did contain compounds with reducing properties, it was quite logical to use them for the green synthesis of gold and silver NPs. Indeed, such a synthesis was successfully carried out, which was confirmed by a TEM study. At the same time, gold and silver NPs differed in both shape and size. In addition, all solutions of AuNPs were found to be unstable, which was different from the colloidal solutions of AgNPs, which remained stable for a long time.

We consider it to be important that after 7 days gold NPs obtained using extracts from the roots of the control plants or hairy roots differed in the degree of sedimentation. In particular, the smallest sedimentation was observed in samples of AuNPs obtained using the roots of the control samples (Fig. 3, AuNPs flask 4). Comparatively, the composition of the extracts, shown in Table 1, indicates a significantly higher content of simple phenols and derivatives of hydroxybenzoic acids in the transgenic roots than in the control. It can be assumed that a high concentration of these components can negatively affect the stability of colloidal solutions of gold NPs but did not affect the stability of silver NPs. According to MALDI-MS study (Tables 2 and 3) the content of such components as quinic and chlorogenic acids in the extract from the control roots was greater than in transgenic one. Beside this, the smaller content of caffeic acid derivatives (OC-, OC-4, and OC-6) also may cause the quick sedimentation since it was the NPs line No 2-14 that had the greatest tendency to precipitate. These components were studied earlier as stabilizers of metal NPs (Guo et al., 2015). The possibility of such an assumption is evidenced by the fact that the highest concentration of OC components was found precisely in the extract from the roots of the control plants. The colloidal solution of AuNPs obtained using this extract was the most stable and was stored for more than 7 days.

The differences in the stability of AgNPs and AuNPs are probably due to the fact that the process of formation of AgNPs was ‘stretched’ in time. This is evidenced by the dynamics of changes in the UV-Vis spectra (Fig. 1C). A larger number of formed NPs can lead to the prevalence of the sedimentation process over other processes in the colloidal solution and a violation of the sedimentation–diffusion equilibrium (Giorgi et al., 2021). In addition, the significant increase in the size of the AuNPs, as shown above (Fig. 4B), could have contributed to the instability of the colloidal solution of AuNPs. This also likely contributed to the imbalance and has led to the sedimentation of the NPs.

The role of flavonoids as mediators in metal NPs synthesis due to their H+ donating capability was studied earlier (Sathishkumar et al., 2018). The bioreduction potential of different phenolic acids was related to the number of functional hydroxyl groups in the molecules (Scampicchio et al., 2006). For example, caffeic acid acted as reducing agent and stabilizer and was used for AgNPs (Guo et al., 2015) and AuNPs (Seo et al., 2017) synthesis. Chlorogenic acid demonstrated the similar activity in the process of obtaining silver and gold NPs (Noh et al., 2013; Hwang et al., 2015).

The sizes of silver NPs that were obtained using two different hairy root extracts did not differ (Fig. 4A). At the same time, significant differences in the sizes of NPs obtained using the extract from the control roots were found (statistical significance was marked as a and b). A similar situation was observed in the analysis of gold NPs. Such differences could be caused by the above-described peculiarities of the chemical composition of the used extracts. At the same time, it should be noted that silver and gold NPs obtained using the same extract (it can be both the extract from transgenic roots and an extract from the control roots) have significant differences in size (statistical significance was marked as A, B, and C). The median diameters of freshly obtained AuNPs were 2.8-, 3.5-, and 1.3-fold less than the diameters of correspondent AgNPs.

On the other hand, storage for 2 weeks resulted in 5.7- and 6.8-fold size-increase for AuNPs obtained by hairy root lines 2-3 and 2-14 extracts, respectively. Statistically significant changes in the median diameter were not noticed, but particle size redistribution was observed in the case of both transgenic and control roots. Thus, the share of AuNPs smaller than 5 nm decreased to 11%, 6%, and 9%. Particles larger than 75 nm appeared. Such a significant increase in the size of NPs can be the cause of sedimentation under the condition of insufficient concentration of stabilizing agents in the used extracts. Thus, it is likely that engineered NPs obtained using different extracts have their own properties, which can largely be related to the chemical composition of the extracts used.

Green synthesized NPs were studied in the reaction of MB photodegradation. The same process was studied earlier with different NPs, such as WO_3_NPs (Yin et al., 2021), AuNPs (Maliszewska et al., 2021; Suvith and Philip, 2014), AgNPs (Suvith and Philip, 2014). In our experiments great differences in MB photodegradation were detected in case of gold or silver NPs using in the reaction. The catalytic activity of AuNPs regarding MB degradation was detected; at the same time AgNPs did not demonstrate such activity because no changes in the characteristic absorption peak were observed in this case. Differences in catalytic efficiency of AgNPs and AuNPs were demonstrated earlier (Worakitjaroenphon et al., 2023). AuNPs were 2-fold more active in the reaction of 4-nitrophenol reduction than AgNPs obtained by the same method.

It is worth noting that a recently published study (Ahmad et al., 2024) showed the possibility of AgNPs and AuNPs obtaining using a crude plant extract of *Aconitum violaceum*. The authors did not find crucial differences in the level of MB photodegradation when AgNPs or AuNPs were used, which differs from our results. The lack of specific activity of AgNPs is possibly the result of the characteristics of NPs when using a particular extract (different extract compositions can cause differences in the number, shape, and activity of NPs). In addition, these differences may arise due to application of different methods of NPs obtaining and such parameters of the process as temperature, formation time, pH of the mixture, etc.

## MATERIALS AND METHODS

### Plant material and extracts preparation

*A. annua* hairy roots lines 2-3 and 2-14 from the collection of the Laboratory of Adaptational Biotechnology of the Institute of Cell Biology and Genetic Engineering of NAS of Ukraine obtained earlier were subcultivated on solidified Murashige and Skoog (Duchefa, Netherlands) medium over 10 years (Fig. 1A). For extracts preparation the roots were grown in the liquid MS medium for 3 weeks, washed with deionized water, frozen in Brunswick scientific ultra-low temperature freezer U570 (–70°C), lyophilized in Labconco freeze dryer 4.5, powdered (Retsch MM400, Germany), extracted with 70% ethanol for 48 h (root weight, g; ethanol volume, ml, 1:30), and filtered using Whatman filter paper. Control plants were grown under the same conditions. Extracts were collected and standardized to a concentration of 1 mg/ml in rutin equivalent (RE).

### Total flavonoid content assay

Total flavonoid content was studied spectrophotometrically (Fluorat-02 Panorama, λ=510 nm) by modified method (Pekal and Pyrzynska, 2014) using 70% ethanol plant extracts. For this purpose, 0.25 ml of each extract was mixed with 1 ml of deionized water and 0.075 ml of 5% NaNO_2_ solution. In 5 min 0.075 ml of 10% AlCl_3_ solution was added to the mixture. After 5 min of incubation at 24°C, 0.5 ml of 1 M NaOH solution and 0.6 ml of deionized water were added to the reaction mixture. Calibration plot C_rutin_=2.4408×(R^2^=0.9630) was applied to calculate the flavonoid content in rutin equivalent (mg RE/g).

### Reducing power assay

Reducing power of the extracts was studied by their ability to reduce Fe3^+^ to Fe2^+^ (modified from the method described in Zhao et al., 2008). Ethanol extracts (0.016-0.125 ml) were added to 0.3 ml of phosphate buffer (pH 6.6) and 0.3 ml of potassium ferricyanide (1%), mixed thoroughly and incubated at 50°C for 30 min. After this procedure, 0.3 ml of trichloroacetic acid (10%) was added. Then 1.25 ml of the solution was mixed with 1.25 ml of double-distilled water and 0.25 ml of FeCl_3_ (0.1%). Absorbance was spectrophotometrically studied at 700 nm (Fluorat-02 Panorama). The curves of change in optical density were constructed for each extract. Reducing power expressed as equivalent concentrations (EC_0.5_) was determined as the amount of dried root material needed to obtain absorbance (A700) 0.5. Rutin solution (1 mg/ml) was used as a positive control.

### Preparation of AgNPs and AuNPs

The aqueous solution of 1 mM silver nitrate (AgNO_3_, Sigma) was prepared in a 100 ml flask. The ethanol root extract (0.02, 0.04, and 0.08 ml) was mixed with 1 ml of AgNO_3_ solution under magnetic stirring at 90°C during 1 h for the reduction of Ag^+^ to Ag^0^ and then was left at 28°C until the colour of the mixture changed from yellowish to brown.

For gold NPs synthesis the extracts (0.02, 0.04, and 0.08 ml) were added to the aqueous solution of 2 mM HAuCl_4_ (Sigma). The mixture was heated at 50°C for 30 min until the colour of the mixture changed from yellowish to pink.

### Characterisation of NPs

TEM was used to examine the size and morphology of synthesized NPs. The images were captured with a JEM-1400 (Jeol, Japan) microscope with accelerating voltage of 80 kV. The samples were prepared by applying a suspension of NPs to a copper grid with a formvar coating, which is reinforced by carbon sputtering. We used copper grids with combined coating because such films are more stabilized under the electron beam. Moreover, the carbon layer provided better electrical and thermal conductivity of the electron beam from the sample to the grid bars. Finally, there was little to no specimen drift due to charge and thermal effects of the electron beam. The latter was important for a better visualization of the sample image.

The sizes of NPs were measured by ImageJ software (https://imagej.net/ij/download.html) using TEM photos. Areas of particles were measured instead of diameters due to non-spherical shapes of some particles. Results were calculated and presented as nominal diameters.

FTIR spectra were obtained using a Nicolet IS50 FTIR spectrometer equipped with a built-in attenuated total reflection (ATR) system with a diamond crystal (Thermo Fisher Scientific, USA). FTIR spectra were recorded in the range of wave numbers of 4000-400 cm^-1^ with 16 scans with a resolution of 4 cm^-1^.

UV-visible method was employed for the complex analysis of biosynthesized colloid solutions of NPs. The spectra of the colloidal solution (surface plasmon resonance, SPR) were measured periodically (1, 7 and 14 days) in the wavelength range 300-700 nm by Fluorat-02 Panorama spectrophotometer (PanoramaPro software). In some samples the content of obtained solution was diluted by deionized water for measurements.

### Photocatalysis of MB solution by NPs

The study was performed using UV-Vis spectroscopy (Fluorat-02-Panorama spectrofluorimeter, PanoramaPro software) according to method described by León et al. (2016). Each sample of AuNPs solution (0.75 ml) was mixed with MB aqueous solution (15 ml, 0.01%) in transparent glass beakers and put under UV-lamp (36W, 365 nm) to initiate catalysis. With constant stirring, every 15 min the volume of the MB solution was withdrawn in order to obtain its UV-Vis absorption spectrum (500-800 nm). The same procedure was repeated with the blank MB solution. This protocol was also used to study the effect of AgNPs in the reaction with MB.

### Chromatographic analysis

Before analysis, the extracts were filtered through a filter with a hydrophilic nylon membrane with pores of 0.22 μm. The analysis was carried out by the HPLC method on the Agilent 1260 InfinityII system with a diode-matrix detector and a Poroshell 120 EC-C18 column (3.0×150 mm, 2.7 μm). An aqueous solution of 0.05 M H_3_PO_4_ was used as eluent A, and acetonitrile was used as eluent B. The sample volume was 2 μl, the column temperature was 30 °С. Detection was carried out at wavelengths of 206 nm, 300 nm and 350 nm.

### Mass spectrometric analysis

Matrix-assisted laser desorption/ionization time-of flight mass spectrometry (MALDI MS) has been used to study the composition of three samples of *A. annua* root extracts. Positive- and negative-ion mass spectra were recorded using an Autoflex II (Bruker Daltonics) instrument with nitrogen laser (337 nm). For sample preparation, 0.5 μl of a THAP matrix [saturated solution of 2,4,6-trihydroxyacetophenone (Sigma) in acetone] was pipetted on a steel target followed by depositing 1 μl of analyte sample. After air-drying, the target was mounted into an analyzing system of a mass spectrometer, and the deposited samples were subjected to pulse laser irradiation (pulse duration 3 ns, frequency 20 Hz). Spectra were registered under linear detection mode with a 10 ns ion extraction delay and an accelerating voltage of 20 kV. Final mass spectra were the sum of 10 spectra accumulated via irradiation with 10 laser pulses at each individual point on the target with the sample deposited. The laser power was chosen as such providing an optimal signal-to-noise ratio and was kept the same for all the samples studied. The obtained mass spectra treatment and analysis were performed using a FlexAnalysis (Bruker Daltonics) software. After excluding obvious matrix-related ions, the assignment of the most probable compounds relating to the analyte ions in the mass spectra was performed taking into account own HPLC results, the literature data on the possible constituents of the extracts from *Artemisia* species (Anibogwu et al., 2021; Bordean et al., 2023; Ekiert et al., 2022), as well as via referring to the on-line metabolomics database (https://www.metabolomicsworkbench.org/search/ms.php). The ion was considered as matched if the deviation in its main monoisotopic mass from the theoretical one was not higher than 0.1 Da.

### Statistical analysis

Statistical analysis and some graphing were performed using the R (version 4.4.1) software. Resulted values were performed as mean±standard error or median. The data were tested on normality by the Shapiro–Wilk test and homogeneity of variance by Levene's test. Comparisons were performed by ANOVA, followed by the Tukey HSD test, or the Kruskal–Wallis rank sum test, followed by the corresponding multiple comparison test from the pgirmess package. *P*-values less than 0.05 were considered significant.

### Conclusions

The research results indicate the possibility of using not only extracts from *A. annua* plant roots, but also transgenic plant roots (hairy roots) of this species for the synthesis of AuNPs and AgNPs. The extracts from the control and hairy roots used for NPs green synthesis differed in the content of simple phenols and derivatives of hydroxybenzoic acids, terpenoids, caffeic and chlorogenic acids. Probably, such differences affected the process of AuNPs and AgNPs formation and their parameters. Solutions of oval and rounded AgNPs were characterized by high stability for a long time. In contrast, gold NPs aggregated within a short time, increasing in size, thus contributing to the instability of the colloidal solution.

## Supplementary Material

10.1242/biolopen.061739_sup1 Supplementary information
