# Supplementary material for: Comparison of silver and gold nanoparticles green synthesis by Artemisia annua hairy root extracts
Source: Biol Open. 2025 Mar 19;14(3):bio061739. doi: 10.1242/bio.061739 (PMC11957450; doi:10.1242/bio.061739)
Supplement: Supplementary information [file biolopen-14-061739-s1.pdf]

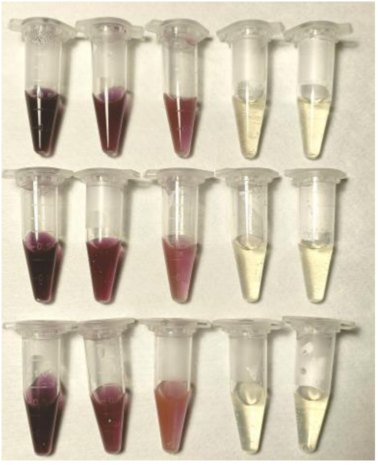

Fig. S1. Dependence of AuNPs initiation on the content of *Artemisia annua* root extracts in the reaction mixture. Extracts of the control roots (cr), hairy root lines NoNo 2-3 and 2-14 were used in the ratio of 5 … 80 μL/1 mL for green synthesis of NPs.

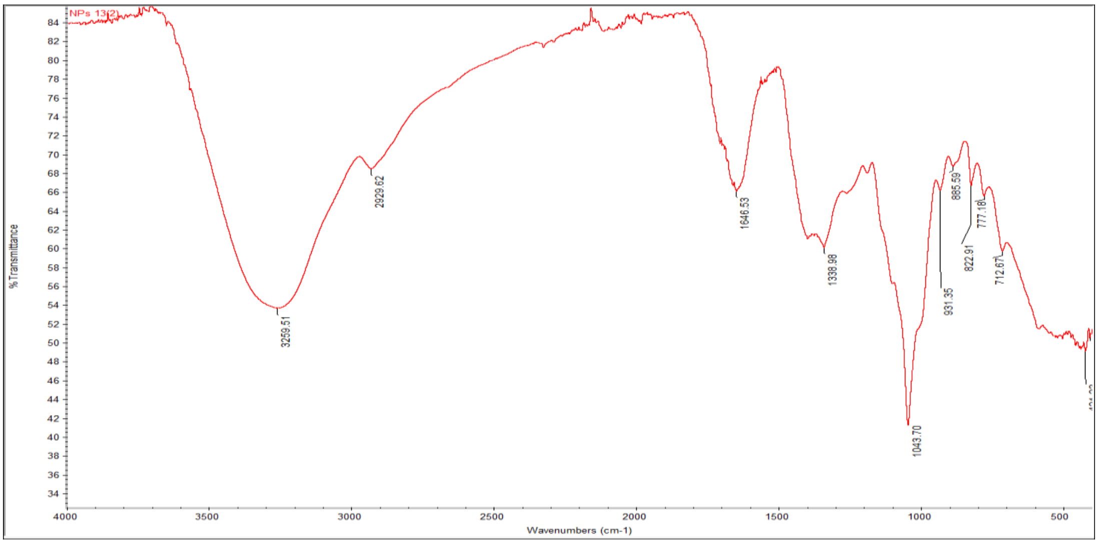

Fig. S2. FTIR spectrum of AgNPs synthesized using extracts of *A. annua* control roots.

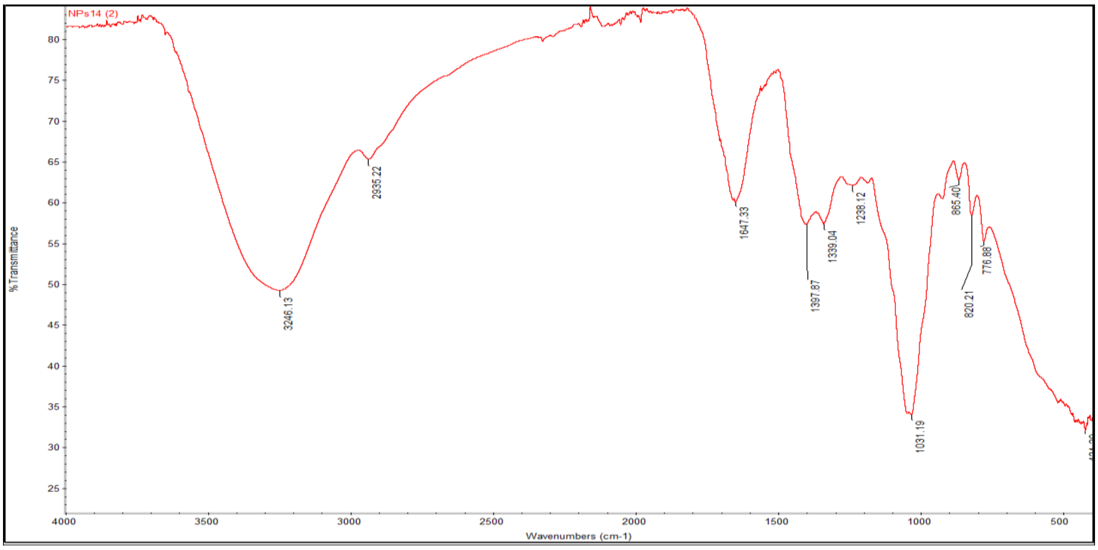

Fig. S3. FTIR spectrum of AgNPs synthesized using extracts of *A. annua* hairy roots No 2-3.

Biology Open • Supplementary information

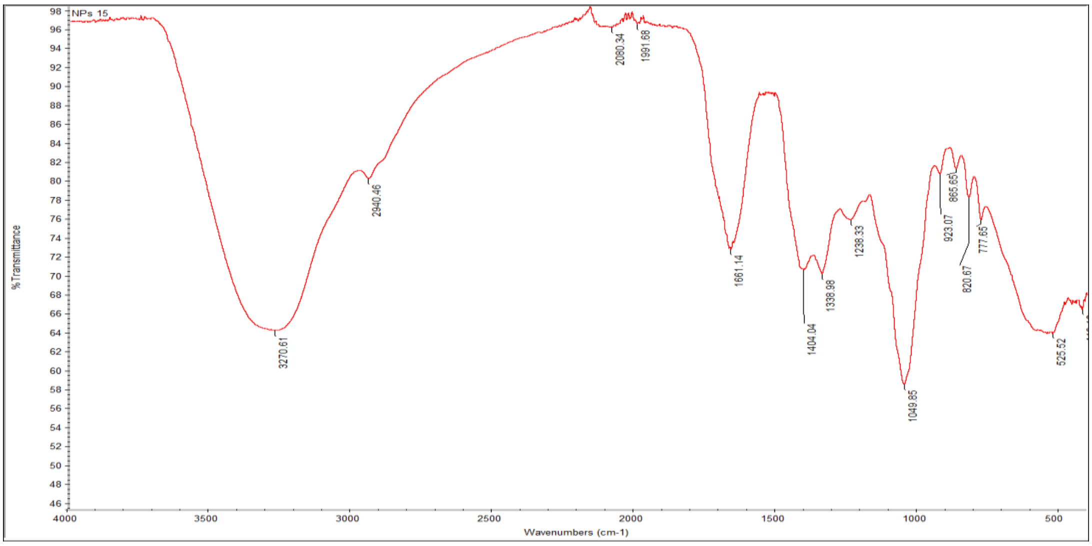

Fig. S4. FTIR spectrum of AgNPs synthesized using extracts of *A. annua* hairy roots No 2-14.

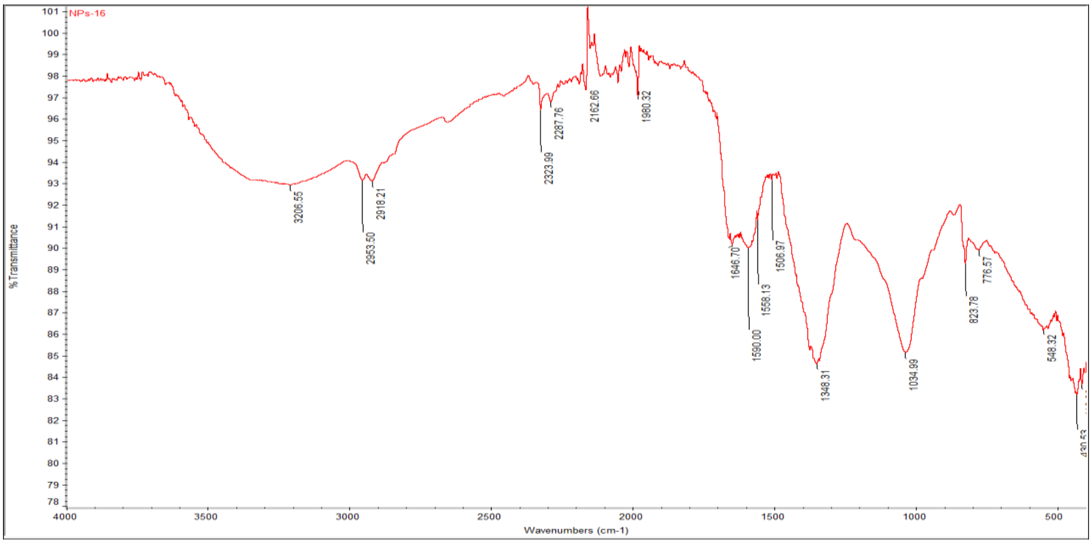

Fig. S5. FTIR spectrum of AuNPs synthesized using extracts of *A. annua* control roots.

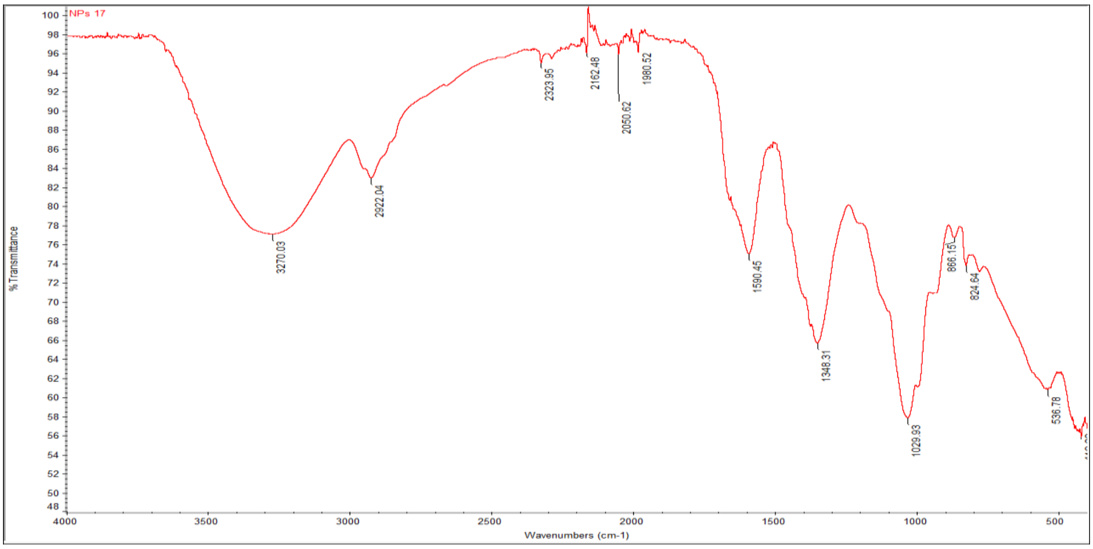

Fig. S6. FTIR spectrum of AuNPs synthesized using extracts of *A. annua* hairy roots No 2-3.

Biology Open • Supplementary information

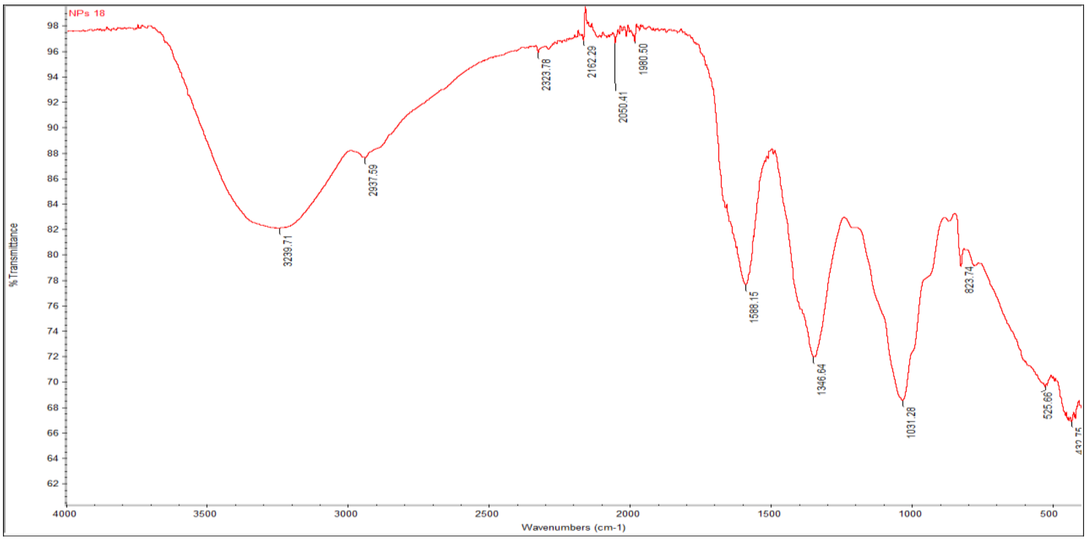

Fig. S7. FTIR spectrum of AuNPs synthesized using extracts of *A. annua* hairy roots No 2-14.

Biology Open • Supplementary information
